# Prevalence of drug–drug interaction in atrial fibrillation patients based on a large claims data

**DOI:** 10.1371/journal.pone.0225297

**Published:** 2019-12-09

**Authors:** Kenji Momo, Haruna Kobayashi, Yuuka Sugiura, Takeo Yasu, Masayoshi Koinuma, Sei-ichiro Kuroda

**Affiliations:** 1 Department of Pharmacy, The Institute of Medical Science Hospital, The University of Tokyo, Minato-ku, Tokyo, Japan; 2 Faculty of Pharmaceutical Sciences, Teikyo Heisei University, Nakano-ku, Tokyo, Japan; 3 Department of Hospital Pharmaceutics, School of Pharmacy, Showa University, Shinagawa-ku, Tokyo, Japan; National Chiao Tung University College of Biological Science and Technology, TAIWAN

## Abstract

This study aimed to compare and determine the prevalence of drug–drug interaction (DDI) and bleeding rate in atrial fibrillation (AF) patients receiving anticoagulants in a clinical setting. We used large claims data of AF patients obtained from the Japan Medical Data Center. The prevalence of DDIs and cases leading to bleeding events were surveyed clinically relevant DDIs extracted from 1) reported from a spontaneous adverse event reporting system (Japanese Adverse Drug Events Report system; JADER) ≥4 patients; 2) DDIs cited in the package inserts of each anticoagulant (each combination assessed according to “Drug interaction 2015” list; 3) warfarin and quinolone antibiotics DDIs. DDIs were categorized the mechanisms for pharmacokinetic DDI (Cytochrome P450 (CYP) or transporter etc. that modulate blood concentration of anticoagulants)/pharmacodynamic DDI (combination with similar pharmacological actions) or both in the analysis for each patients’ prescriptions obtained from a claims data. AF patients were compared between cases with and without bleeding after administered of anticoagulants. Bleeding was observed in 220/3290 (6.7%) AF patients. The bleeding rate in patients with both pharmacokinetic and pharmacodynamic DDI mechanisms (26.3%) was higher than that in patients with either mechanism (8.6% and 9.2%, respectively) or without DDIs (4.9%). The odds ratio for bleeding in AF patients with both of pharmacokinetic and pharmacodynamic was (7.18 [4.69–11.00], p<0.001). Our study concluded multi mechanism based DDIs leads serious outcome as compared to that of single mechanism based DDIs in AF patients. We determined the prevalence and frequency of bleeding for anticoagulant-related DDIs. To manage DDIs, both pharmacokinetic and pharmacodynamic DDI mechanisms should be closely monitored for initial symptoms of bleeding within the first 3 months.

## Introduction

In the present decade, the number of patients with atrial fibrillation (AF) has gradually increased in parallel with the extended lifespan [1]. The treatment of AF includes rhythm control and prevention of thrombosis using anticoagulants, such as warfarin or direct oral anticoagulants (apixaban, edoxaban, dabigatran, and rivaroxaban). These anticoagulants are known to exhibit pharmacodynamic DDIs with antiplatelet drugs and pharmacokinetic DDIs with cytochrome P450 (CYP) or transporter inhibitors, inducing bleeding events.

A retrospective survey reported that 26% of adverse events for direct hospital admissions were caused by drug–drug interactions (DDIs) [2]. The prevalence of potential DDIs was commonly observed among inpatients (19%) and outpatients (31%) in large scale observational studies [3,4]. DDIs, including those between anticoagulant and antiplatelet drugs, frequently led to bleeding in 19.4% cases of double anticoagulant therapy and 44.4% cases of triple anticoagulant therapy in the first observational year [5]. In addition, the co-administration of anticoagulants, azole antifungals, and amiodarone increased the risk of major bleeding [6]. However, data on these situations, including those on any bleeding in real-world clinical settings, are insufficient because of the limited case reports and pharmaceutical information. The number of cases with DDIs reported is generally insufficient in hospital or community pharmacies.

Recently above mentions problem, some medical big data such as claim data or spontaneous adverse events reporting systems were applying to the researches. In Japan, a large health insurance claims data has been developed by the Japan Medical Data Center (JMDC) Co. Ltd., Tokyo, Japan. JMDC collects medical and pharmacy claims from >50 occupation-based public health insurance agencies for corporation employees and their family members. As of August 2016, these data included 3,600,000 recipients aged 0–74 years, representing 2.0% of the total Japanese population [7]. In addition, the Japanese Adverse Drug Events Report (JADER), a web-based spontaneous adverse events information collecting system and an open data source in Japan, by the Pharmaceuticals and Medical Devices Agency (PMDA) has been widely used for research [8]. JADER has collected data on >300,000 cases of spontaneous adverse events since 2003. These cases have been entered into the database by physicians, pharmacists, and other medical staff.

In this study, the prevalence of DDIs and bleeding rate was determined based on large claims data for the combination of anticoagulant related clinically relevant DDIs that including a spontaneous adverse event reporting system (Japanese Adverse Drug Events Report system; JADER) in AF patients. The frequency of bleeding without and with DDIs, caused by anticoagulants, was compared. Thus, we aimed to determine the prevalence of anticoagulant-related DDIs in real-world clinical settings.

## Materials and methods

### Identification of anticoagulants related clinically relevant DDI in AF patients

The protocol for this observational study was approved by the ethics committee of Teikyo Heisei University.

We defined the clinically relevant DDIs was as follows 1) Reported ≥4 patients in JADER; 2) DDIs cited in the package inserts of each anticoagulant (each combination assessed according to “Drug interaction 2015” list [9]); 3) Warfarin and quinolone antibiotics. And we excluded DDIs for” unknown mechanism”, “not cited in the package inserts of anticoagulants” and “NSAIDs that independently CYP inhibitor with warfarin DDI”. The pharmacokinetic and pharmacodynamic mechanism was assessed for each DDI.

**Fig 1:**
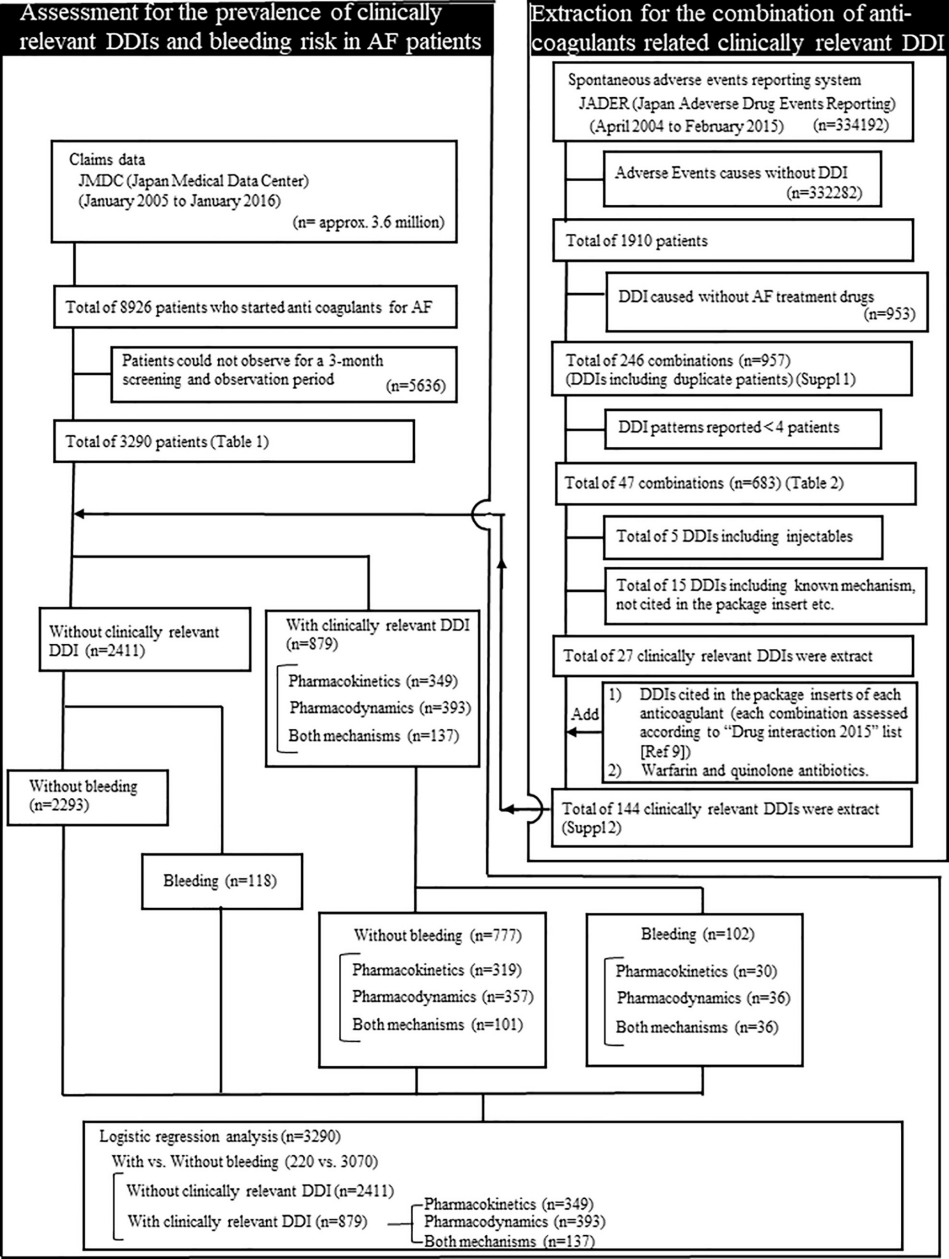
Case identification flow.

Briefly, we searched the JADER database from April 2004 to February 2015 (334192 records, downloaded in 2015) (Fig 1). Patients with adverse events that were caused by suspected DDIs (keywords “interaction” and “suspected drugs for interaction”) were queried to the database. Data collected from JADER included outcome and co-administered drugs. The outcomes of DDIs were classified as severe (death/sequelae/non-recovery), non-severe (remission/recovery), and unknown. Total of 246 DDIs was observed (S1 Table). The above mentioned clinically relevant DDIs full list was shown in S2 Table.

### Prevalence of DDIs and bleeding rate in patients treated for AF in the large claims data

For determining the prevalence of potential DDIs and bleeding events, claims data on 8926 patients diagnosed with AF were used; all patients were administered anticoagulants between January 2005 and January 2016. A retrospective cohort study was performed with a 3-month screening and observation period to determine the prevalence of clinically relevant DDIs and the frequency of bleeding.

A total of 3290 (male/female: 2754/536; 50.1 ± 10.3 years) patients who satisfied the inclusion criteria (3-month screening and observation periods and no missing data) were enrolled in this study. The anticoagulants used by these patients were warfarin (n = 1,208), apixaban (n = 408), edoxaban (n = 21), dabigatran (n = 850), and rivaroxaban (n = 803) (Table 1).

**Table 1:** Characteristics of patients with atrial fibrillation (from the claims data).

Number of patients		3290
Age [mean (SD)]		50.1 (10.3)
Sex (male) [n (%)]		2754 (83.7)
Sex (female) [n (%)]		536 (16.3)
Anticoagulant [n]		
	Warfarin	1208
	Apixaban	408
	Edoxaban	21
	Dabigatran	850
	Rivaroxaban	803
Comorbidities (ICD-10) [n]		
	Hypertensive diseases	1421
	Diabetes mellitus	1386
	Esophagus, stomach, and duodenum diseases	1218
	Liver diseases	796

The adverse events for bleeding were identified using the target word for “bleeding” or “hemorrhage” in “Japanese standard disease master” in this study within 3 months after starting anticoagulants. Detected all bleeding events was shown in S3 Table. This code was linked to ICD-10 code. The standard disease master was developed by “The committee for Controlled Medical Terminology of Japan Association of Medical Sciences” with the responsibility for standardizing disease names, and another committee dedicated for assigning codes to the unique disease names. This is set up in the Social Insurance Medical Fee Payment Fund together with Medica Information System Development Center [10]. Ministry of Health, Labour and Welfare indicated "Japanese standard disease master" as the standard code and inform and use in Japan. This master contains about 22000 terms and 2000 modifiers.

### Statistical analysis

Data were expressed as mean ± standard deviations. For comparison, the frequency of bleeding was assessed between the groups (without and with clinically relevant DDIs) using the chi-squared test. The factors observed p<0.05 in the univariate analysis were add to the multivariate logistic regression models for calculate odds ratios (95% confidence intervals: 95% CI). Variables considered in the model were sex, age, DDI mechanism for pharmacokinetics, pharmacodynamics and both mechanism.

All tests were two-sided and statistical significance was considered to be established at a p-value of less than 0.05. The data analysis was used JMP 14^®^ (with superscript R) (SAS 146 Institute Inc., NC, US).

## Results

### Extraction for the combination of anti-coagulants related clinically relevant DDIs from JADER reported DDIs

A total of 1910 (male/female: 1004/906) patients reported in JADER (n = 334192) were assessed for DDIs. Anticoagulant-related DDIs reported for ≥ 4 cases consist of 47 drug combinations (Fig 1, Table 2). The most frequent combination was between miconazole and warfarin (n = 114). The outcomes provided from the database were severe (n = 7), non-severe (n = 96), and unknown (n = 11).

**Table 2:** Number of patients with anticoagulant-related drug–drug interactions in ≥4 cases reported in JADER.

No.	Drug	Drug	n	The number of patients co-administered with other potentially interaction drug	Potentially interacting drugs						Death/Sequelae/Non-recovery	Remission/ Recovery	Un known	Drug interaction 2015 (mainly PK DDIs)
					Anti-coagulant	Anti-cancer agent	CYP inhibitor	Anti-biotics	Unknown mechanism	Menatetrenone				
1	Miconazole	Warfarin	114	16	3	10	2	4	1	-	7	96	11	☑
2	Rivaroxaban	Aspirin	94	14	16	-	-	-	-	-	28	62	4	□
3	Tegafur–gimeracil–oteracil	Warfarin	78	9	8	-	-	-	-	-	3	73	2	☑
4	Aspirin	Warfarin	34	32	9	9	17	3	4	-	3	28	3	□
5	Clopidogrel	Rivaroxaban	27	14	16	-	-	-	-	-	7	18	2	□
6	Capecitabine	Warfarin	25	8	1	7	-	-	-	-	2	20	3	☑
7	Tegafur–uracil	Warfarin	24	6	2	1	3	-	-	-	2	21	1	☑
8	Bucolome	Warfarin	20	10	7	-	9	-	-	-	1	17	2	☑
9	Loxoprofen	Warfarin	18	14	5	3	3	3	2	-	2	14	2	□
10	Celecoxib	Warfarin	17	0	-	2	1	-	3	-	0	15	2	☑
11	Lansoprazole	Warfarin	16	0	-	-	2	-	-	-	0	7	9	□
12	Allopurinol	Warfarin	10	8	3	-	6	-	3	-	0	10	0	□
13	Levofloxacin	Warfarin	10	5	2	-	2	1	1	-	1	9	0	□
14	Amiodarone	Warfarin	9	2	2	-	-	-	-	-	1	8	0	☑
15	Tazobactam-piperacillin	Warfarin	9	2	-	-	-	4	-	-	1	8	0	□
16	Lornoxicam	Warfarin	9	4	4	-	2	-	-	-	1	8	0	□
17	Clarithromycin	Warfarin	8	6	5	-	5	1	-	-	1	6	1	□
18	Benzbromarone	Warfarin	8	1	1	-	-	-	-	-	1	7	0	□
19	Azithromycin	Warfarin	7	6	1	1	2	1	1	-	0	7	0	□
20	Oxaliplatin	Warfarin	7	7	-	10	-	-	-	-	1	5	1	□
21	Omeprazole	Warfarin	7	3	1	-	-	-	3	-	1	6	0	□
22	Tramadol-Acetaminophen	Warfarin	7	2	2	-	2	-	1	-	0	3	4	□
23	Folinate	Warfarin	7	7	-	7	1	-	-	-	0	7	0	□
24	Erlotinib	Warfarin	6	1	1	1	-	-	-	-	0	5	1	□
25	Fluorouracil	Warfarin	6	2	-	2	-	-	-	-	0	4	2	☑
26	Fluconazole	Warfarin	6	2	-	-	2	1	-	-	1	4	1	☑
27	Rosuvastatin	Warfarin	6	1	-	1	-	-	-	-	0	5	1	□
28	Iguratimod	Warfarin	5	1	-	1	-	-	-	-	1	4	0	□
29	Gliclazide	Warfarin	5	5	5	-	11	-	-	-	0	4	1	□
30	Clopidogrel	Warfarin	5	4	3	3	-	-	2	-	0	4	1	□
31	Digoxin	Warfarin	5	5	-	-	3	1	2	-	1	4	0	□
32	Cefoperazone-sulbactam	Warfarin	5	2	1	-	-	1	-	-	0	2	3	□
33	Phenytoin	Warfarin	5	1	-	1	-	-	-	-	0	5	0	☑
34	Sulfamethoxazole Trimethoprim	Warfarin	5	5	1	-	4	-	1	1	2	3	0	□
35	Ticlopidine	Warfarin	5	5	2	1	2	-	1	-	0	4	1	□
36	Bevacizumab	Warfarin	5	5	1	7	-	-	-	-	2	3	0	□
37	Verapamil	Warfarin	5	5	2	1	2	-	1	-	0	4	1	□
38	Voriconazole	Warfarin	5	0	-	-	-	-	-	-	0	5	0	□
39	Garenoxacin	Warfarin	5	2	1	-	3	1	-	-	0	4	1	□
40	Regorafenib	Warfarin	5	1	1	-	-	-	-	-	0	4	1	□
41	Prednisolone	Warfarin	5	4	3	-	2	-	-	-	1	3	1	□
42	Ciprofloxacin	Warfarin	4	1	-	-	-	1	-	-	0	3	1	□
43	Ezetimibe	Warfarin	4	0	-	-	-	-	-	-	0	4	0	□
44	Carbamazepine	Warfarin	4	0	-	-	-	-	-	-	0	2	2	☑
45	Quetiapine	Warfarin	4	2	1	-	2	-	-	-	0	4	0	□
46	Pranlukast	Warfarin	4	4	4	-	8	-	-	-	0	4	0	□
47	Minocycline	Warfarin	4	2	-	-	3	-	-	-	2	2	0	□

We finally defined total of 144 clinically relevant DDIs from 1) above mentioned DDIs from JADER (finally 27 DDIs from 47 DDIs); 2) DDIs cited in the package inserts of each anticoagulant (each combination assessed according to “Drug interaction 2015” list [Ref 9]); 3) Warfarin and quinolone antibiotics.

### Prevalence and bleeding rate for 144 clinically relevant DDIs in the claims data

The prevalence of 144 Clinically relevant DDIs was analyzed with a 3-month screening and observation period using a large claims data (Fig 1).

Miconazole and warfarin combination therapy was the most frequently reported DDI in JADER; however, patients treated with miconazole and warfarin were not observed in our retrospective cohort in the 3-month observational period using the claims data (S4 Table). Conversely, aspirin and warfarin combination therapy was the most frequent in the claims data (n = 252) observed in 7.7% patients with AF. Bleeding events were observed in 56 (22.2%) of these patients within 3 months of starting warfarin therapy. Patients with existing DDIs were 879/3290 (26.7%) of 144 combinations (Fig 1, S2 and S4 Tables). Bleeding was observed in 220 patients of total 3,290 patients (6.7%) (Table 3). Bleeding rate was observed among the patients with pharmacokinetic DDI mechanism (8.6%; 30/349). This value was comparable to that of pharmacodynamic DDI mechanisms (9.2%; 36/393). The bleeding rate for patients with both pharmacokinetic and pharmacodynamic DDI mechanisms was 26.3% (36/137). The frequency for bleeding were significantly higher with clinically relevant DDI than that of without clinically relevant DDI (4.9%; 118/2411) in the univariate analysis (p<0.05) (Table 3).

**Table 3:** Factors associates with bleeding after administered anti-coagulant in atrial fibrillation patients in a large claims data in Japan.

			With bleeding (n = 220)	Without bleeding (n = 3,070)	*p* value (univariate analysis)	Adjusted Odds (95% CI)	*p* value (multivariate analysis)
Male, number of patients			165	2,589	Reference	Reference	—
Female, number of patients			55	481	0.0006	1.86 (1.34–2.58)	0.0002
Age, year [SD]			55.1 [11.5]	54.0 [10.2]	0.0502	—	—
Clinically relevant DDIs, number of patients							
		None, %	118 [4.9]	2293 [95.1]	Reference	Reference	—
		Pharmacokinetic mechanism, %	30 [8.6]	319 [91.4]	0.0111	1.74 (1.15–2.65)	0.0095
		Pharmacodynamic mechanism, %	36 [9.2]	357 [90.8]	0.0010	1.96 (1.33–2.90)	0.0007
		Both mechanism, %	36 [26.3]	101 [73.7]	<0.001	7.18 (4.69–11.00)	<0.0001

Patients under poly-DDIs were duplicately counted in each DDI combinations.

To detect the factors, associate to the bleeding were assessed using logistic regression analysis. Factors for sex and DDIs mechanisms for anticoagulation (pharmacokinetic, pharmacodynamic and both) were adjusted (Table 3). The odds ratios for the female sex (1.86 [1.34–2.58], p = 0.0002) was higher than male sex. As compared to without DDI, DDI for pharmacokinetic mechanism (1.74 [1.15–2.65], p = 0.0095), pharmacodynamic mechanism (1.96 [1.33–2.90], p = 0.0007) and both of pharmacokinetic and pharmacodynamic mechanism (7.18 [4.69–11.00], p<0.0001) were observed risk factors associate bleeding in AF patients (Table 3).

## Discussion

In this study, we identified 144 anticoagulant-related DDIs in AF patients using the real-world large claims data. Patients who experienced both pharmacokinetic and pharmacodynamic DDIs had a higher bleeding rate than those without DDIs (26.3% vs. 4.9%). Patients with both mechanism of DDI had the higher odds ratio for bleeding (7.18 [4.69–11.00], p<0.0001).

JADER data are useful for identifying DDIs that were focused by clinicians. Several methods for analyzing signal detection of DDIs using reporting odds ratio or proportional reporting ratio have been reported by Nakamura et al. [11, 12] to adjust for the reporting bias. However, it is difficult to calculate the prevalence of DDIs using a spontaneous adverse event reporting system. Therefore, we conducted this study using both JADER and the claims data.

The most frequently reported combination in JADER was between warfarin and miconazole (n = 114/1910 reports), in which miconazole is known to inhibit cytochrome P450 2C9 [13–15]. This combination is known to increase bleeding risk as several case reports have been confirmed till date [14]. Accordingly, in November 2016, warfarin and miconazole combination therapy was announced as a contraindication by pharmaceutical companies and PMDA in Japan [16]. This combination was, however, not observed in the real-world data (our study patient data obtained from January 2005 to January 2016). Our dataset is including the patients aged ≤ 74 years. The prevalence for oral candida is generally observed with lower physical conditions such as elderly patients in Japan [17]. In our JMDC database patients are mainly focused working age population and their family members. This database depending age related bias affects the results that the combination for warfarin and miconazole was not observed in our data (Table 2, S4 Table).

In general, pharmacodynamic DDI mechanism related to antiplatelets in AF patients were frequently observed in the clinical setting. The bleeding rate in our study was comparable to that reported by a previous study that determined the rate to be 10% in anticoagulant double therapy (S4 Table), 30% at 3 months after starting the combination therapy in triple therapy, and 7%–15% in patients who underwent percutaneous coronary intervention [5,18]. These anticoagulant-related DDIs occurred during standard therapy in AF patients who underwent percutaneous coronary intervention, for whom strict control using double or triple anticoagulant therapy for several months was necessary [5,19]. In general, to manage typical DDIs in clinical settings, the pharmacokinetic mechanism via cytochrome P450 inhibition/induction of drug combinations can be predicted by detecting changes in blood concentration and clinical impact [20–22]. However, management of pharmacodynamic DDIs is not easy as there are large inter-individual variations in drug response. Therefore, AF patients with potential pharmacodynamic DDIs need to be monitored for initial bleeding symptoms, especially patients receiving 4 pharmacodynamic DDIs (rivaroxaban–aspirin, warfarin–aspirin, rivaroxaban–clopidogrel and warfarin–clopidogrel), which were focused on by clinicians and were frequently observed in the claims data (Table 2, S4 Table).

In this study, bleeding rate due to combined pharmacokinetic and pharmacodynamic DDI mechanisms was high compared with that due to either pharmacokinetic or pharmacodynamic mechanism alone or that in patients without DDI (Table 3). This finding suggests that a multi-mechanism DDI has a higher risk than a simple mechanism for two-drug DDI in patients with AF. Physicians as well as pharmacists need to monitor patients undergoing percutaneous coronary intervention for bleeding as these patients tend to show a multi-mechanism DDI.

JADER is based on the reporting of spontaneous adverse events from medical staffs. This database could not calculate the frequency because of lacking of denominator. But the adverse events were clearly cited. JMDC data is based on the claim data. This could calculate the frequency but to detect the adverse events, it needs to definite the definitions to detect the targeted adverse events. JMDC claim database consists of anonymized data on >3.6 million people (inpatients, outpatients and pharmacy claims) aged ≤74 years from ~91.7% of all medical facilities (n = 90,021) in Japan. All patients in JMDC database are take in “social insurance” that including working person and their families. These shows their socioeconomic level have no large differences. Statics for Ministry of Health, Labor and Welfare in 2014 in Japan [23], Japanese subscripted to each medical care system for elderly in the latter stage of life (over 75 years) constituted of pension income (11.4%, average income: 8,300 $/year), national health insurance constituted of most of low- or mid-income person (27.3%, average income: 14,400 $/year), public assistance person with no- or low-insurance (1.6%) and some of employees’ insurance system were exist and that were lower than that of our data population (average income: 38,400 $/year) [23]. In this study, we analyzed using without low income population that means stratified the income and status depending income-related education level. The results suggest that our data reliable for the mid- or high-income AF patients.

## Conclusion

We determined the prevalence and frequency of bleeding due to 144 anticoagulant—related clinically relevant DDIs. Our study concluded multi mechanism based DDIs leads serious outcome as compared to that of single mechanism based DDIs in AF patients. To manage DDIs, patients should be closely monitored for initial symptoms of bleeding within the first 3 months, especially in patients who are likely to experience both pharmacokinetic and pharmacodynamic DDI mechanisms.

## Supporting information

S1 Table DDIs all combination in JADER. (DOCX)

S2 Table Clinically relevant DDIs. (DOCX)

S3 Table The number of patients with bleeding in our study. (DOCX)

S4 Table Prevalence of 42 DDIs from JADER in 3290 patients treated for atrial fibrillation in the large claim data. (DOCX)
