# Supplementary material for: Prevalence of drug–drug interaction in atrial fibrillation patients based on a large claims data
Source: PLoS One. 2019 Dec 9;14(12):e0225297. doi: 10.1371/journal.pone.0225297 (PMC6901225; doi:10.1371/journal.pone.0225297)
Supplement: S1 Table — (DOCX) [file pone.0225297.s001.docx]

| No.  S1 | Drug A | Drug B | The number of patients |  | No. | Drug A | Drug B | The number of patients |
| --- | --- | --- | --- | --- | --- | --- | --- | --- |
| 1 | Warfarin | Miconazole | 114 |  | 31 | Warfarin | Digoxin | 5 |
| 2 | Rivaroxaban | Aspirin | 94 |  | 32 | Warfarin | Cefoperazone-sulbactam | 5 |
| 3 | Warfarin | Tegafur–gimeracil–oteracil | 78 |  | 33 | Warfarin | Sulfamethoxazole Trimethoprim | 5 |
| 4 | Warfarin | Aspirin | 34 |  | 34 | Warfarin | Ticlopidine | 5 |
| 5 | Rivaroxaban | Clopidogrel | 27 |  | 35 | Warfarin | Prednisolone | 5 |
| 6 | Warfarin | Capecitabine | 25 |  | 36 | Warfarin | Bevacizumab | 5 |
| 7 | Warfarin | Tegafur–uracil | 24 |  | 37 | Warfarin | Verapamil | 5 |
| 8 | Warfarin | Bucolome | 20 |  | 38 | Warfarin | Garenoxacin | 5 |
| 9 | Warfarin | Loxoprofen | 18 |  | 39 | Warfarin | Regorafenib | 5 |
| 10 | Warfarin | Celecoxib | 17 |  | 40 | Warfarin | Voriconazole | 5 |
| 11 | Warfarin | Lansoprazole | 16 |  | 41 | Warfarin | Phenytoin | 5 |
| 12 | Warfarin | Levofloxacin | 10 |  | 42 | Warfarin | Ciprofloxacin | 4 |
| 13 | Warfarin | Allopurinol | 10 |  | 43 | Warfarin | Ezetimibe | 4 |
| 14 | Warfarin | Amiodarone | 9 |  | 44 | Warfarin | Carbamazepine | 4 |
| 15 | Warfarin | Tazobactam-piperacillin | 9 |  | 45 | Warfarin | Quetiapine | 4 |
| 16 | Warfarin | Lornoxicam | 9 |  | 46 | Warfarin | Pranlukast | 4 |
| 17 | Warfarin | Clarithromycin | 8 |  | 47 | Warfarin | Minocycline | 4 |
| 18 | Warfarin | Benzbromarone | 8 |  | 48 | Warfarin | Rivaroxaban | 3 |
| 19 | Warfarin | Oxaliplatin | 7 |  | 49 | Warfarin | Aspirin-dialuminate | 3 |
| 20 | Warfarin | Omeprazole | 7 |  | 50 | Warfarin | Acetaminophen | 3 |
| 21 | Warfarin | Tramadol-Acetaminophen | 7 |  | 51 | Warfarin | ‎Atorvastatin | 3 |
| 22 | Warfarin | Folinate | 7 |  | 52 | Warfarin | ‎Amlodipine | 3 |
| 23 | Warfarin | Azithromycin | 7 |  | 53 | Warfarin | Eplerenone | 3 |
| 24 | Warfarin | Erlotinib | 6 |  | 54 | Warfarin | Oseltamivir | 3 |
| 25 | Warfarin | Fluorouracil | 6 |  | 55 | Warfarin | Carvedilol | 3 |
| 26 | Warfarin | Rosuvastatin | 6 |  | 56 | Warfarin | Cefazolin | 3 |
| 27 | Warfarin | Fluconazole | 6 |  | 57 | Warfarin | Cefdinir | 3 |
| 28 | Warfarin | Clopidogrel | 5 |  | 58 | Warfarin | Cefmetazole | 3 |
| 29 | Warfarin | Iguratimod | 5 |  | 59 | Warfarin | Zonisamide | 3 |
| 30 | Warfarin | Gliclazide | 5 |  | 60 | Warfarin | Sorafenib | 3 |
| No. | Drug A | Drug B | The number of patients |  | No. | Drug A | Drug B | The number of patients |
| 59 | Warfarin | Famotidine | 3 |  | 91 | Warfarin | Rabeprazole | 2 |
| 61 | Warfarin | Pregabalin | 3 |  | 92 | Warfarin | Limaprost alfadex | 2 |
| 62 | Warfarin | Moxifloxacin | 3 |  | 93 | Warfarin | Levofolinate | 2 |
| 63 | Dabigatran | Aspirin | 3 |  | 94 | Warfarin | Enteral nutrient (detail unknown) | 2 |
| 64 | Dabigatran | Verapamil | 3 |  | 95 | Warfarin | Enteral nutrient (detail unknown) | 2 |
| 65 | Warfarin | Acarbose | 2 |  | 96 | Warfarin | Fosfluconazole | 2 |
| 66 | Warfarin | Azulene | 2 |  | 97 | Rivaroxaban | Loxoprofen | 2 |
| 67 | Warfarin | Aprepitant | 2 |  | 98 | Rivaroxaban | Aspirin-dialuminate | 2 |
| 68 | Warfarin | Amoxicillin | 2 |  | 99 | Rivaroxaban | Clarithromycin | 2 |
| 69 | Warfarin | Alendronate | 2 |  | 100 | Rivaroxaban | Ethyl icosapentate | 2 |
| 70 | Warfarin | Irinotecan | 2 |  | 101 | Rivaroxaban | Diclofenac | 2 |
| 71 | Warfarin | Esomeprazole | 2 |  | 102 | Rivaroxaban | Limaprost alfadex | 2 |
| 72 | Warfarin | Etodolac | 2 |  | 103 | Dabigatran | Cibenzoline | 2 |
| 73 | Warfarin | Oxycodone | 2 |  | 104 | Dabigatran | Bisoprolol | 2 |
| 74 | Warfarin | Glimepiride | 2 |  | 105 | Warfarin | Dabigatran | 1 |
| 75 | Warfarin | Diclofenac | 2 |  | 106 | Warfarin | Cilostazol | 1 |
| 76 | Warfarin | Cisplatin | 2 |  | 107 | Warfarin | L-cysteine | 1 |
| 77 | Warfarin | Dipyridamole | 2 |  | 108 | Warfarin | Aspirin-dialuminate | 1 |
| 79 | Warfarin | Simvastatin | 2 |  | 109 | Warfarin | Anastrozole | 1 |
| 80 | Warfarin | Sunitinib | 2 |  | 110 | Warfarin | Amitriptyline | 1 |
| 81 | Warfarin | Ceftriaxone | 2 |  | 111 | Warfarin | Amphotericin B | 1 |
| 82 | Warfarin | Pazufloxacin | 2 |  | 112 | Warfarin | Argatroban | 1 |
| 83 | Warfarin | Sodium valproate | 2 |  | 113 | Warfarin | Alprostadil alfadex | 1 |
| 84 | Warfarin | Bicalutamide | 2 |  | 114 | Warfarin | Imatinib | 1 |
| 85 | Warfarin | Fenofibrate | 2 |  | 115 | Warfarin | Indometacin | 1 |
| 86 | Warfarin | Febuxostat | 2 |  | 116 | Warfarin | Exenatide | 1 |
| 87 | Warfarin | Fluvoxamin | 2 |  | 117 | Warfarin | Etoposide | 1 |
| 88 | Warfarin | Furosemide | 2 |  | 118 | Warfarin | Olanzapine | 1 |
| 89 | Warfarin | Bosentan | 2 |  | 119 | Warfarin | Olopatadine | 1 |
| 90 | Warfarin | Mosapride | 2 |  | 120 | Warfarin | ‎Galantamine | 1 |
| No. | Drug A | Drug B | The number of patients |  | No. | Drug A | Drug B | The number of patients |
| 121 | Warfarin | Kallidinogenase | 1 |  | 151 | Warfarin | Nabumetone | 1 |
| 122 | Warfarin | Carbocysteine | 1 |  | 152 | Warfarin | Nifedipine | 1 |
| 123 | Warfarin | Carboplatin | 1 |  | 153 | Warfarin | Norfloxacin | 1 |
| 124 | Warfarin | Clonazepam | 1 |  | 154 | Warfarin | Paclitaxel | 1 |
| 125 | Warfarin | Ketoprofen | 1 |  | 155 | Warfarin | Panipenem-betamipron | 1 |
| 126 | Warfarin | Gemcitabine | 1 |  | 156 | Warfarin | Paroxetine | 1 |
| 127 | Warfarin | Zaltoprofen | 1 |  | 157 | Warfarin | Pioglitazone | 1 |
| 128 | Warfarin | Sarpogrelate | 1 |  | 158 | Warfarin | Pitavastatin | 1 |
| 129 | Warfarin | Diazepam | 1 |  | 159 | Warfarin | Vildagliptin | 1 |
| 130 | Warfarin | Digitoxin | 1 |  | 160 | Warfarin | Vincristine | 1 |
| 131 | Warfarin | Disopyramide | 1 |  | 161 | Warfarin | Faropenem | 1 |
| 132 | Warfarin | Simeprevir | 1 |  | 162 | Warfarin | Fibrinogen combined drug | 1 |
| 133 | Warfarin | Ampicillin-sulbactam | 1 |  | 163 | Warfarin | Fentanyl | 1 |
| 134 | Warfarin | Sulpiride | 1 |  | 164 | Warfarin | Ferrous fumarate | 1 |
| 135 | Warfarin | Cefotiam | 1 |  | 165 | Warfarin | Pravastatin | 1 |
| 136 | Warfarin | ‎Cefcapene | 1 |  | 166 | Warfarin | Fluvastatin | 1 |
| 137 | Warfarin | Cefditoren | 1 |  | 167 | Warfarin | Prochlorperazine | 1 |
| 138 | Warfarin | Ceftazidime | 1 |  | 168 | Warfarin | Propiverine | 1 |
| 139 | Warfarin | Cefpodoxime | 1 |  | 169 | Warfarin | Flomoxef | 1 |
| 140 | Warfarin | Sevoflurane | 1 |  | 170 | Warfarin | PEG-α2 | 1 |
| 141 | Warfarin | General Chemotherapy | 1 |  | 171 | Warfarin | Betamethasone | 1 |
| 142 | Warfarin | Tacrolimus | 1 |  | 172 | Warfarin | Bepridil | 1 |
| 143 | Warfarin | Daptomycin | 1 |  | 173 | Warfarin | Verteporfin | 1 |
| 144 | Warfarin | Tamusulosin | 1 |  | 174 | Warfarin | Fosfomycyn calcium | 1 |
| 145 | Warfarin | Theophylline | 1 |  | 175 | Warfarin | Fosfomycyn sodium | 1 |
| 146 | Warfarin | Terbinafine | 1 |  | 176 | Warfarin | Bortezomib | 1 |
| 147 | Warfarin | Telmisartan | 1 |  | 177 | Warfarin | Extract of ephedra formulation | 1 |
| 148 | Warfarin | Tocilizumab | 1 |  | 178 | Warfarin | Mianserin | 1 |
| 149 | Warfarin | Tramadol | 1 |  | 179 | Warfarin | Micafungin | 1 |
| 150 | Warfarin | Droxidopa | 1 |  | 180 | Warfarin | Mirtazapine | 1 |
| No. | Drug A | Drug B | The number of patients |  | No. | Drug A | Drug B | The number of patients |
| 181 | Warfarin | Mequitazine | 1 |  | 214 | Rivaroxaban | Amiodarone | 1 |
| 182 | Warfarin | Metildigoxin | 1 |  | 215 | Rivaroxaban | Ifenprodil | 1 |
| 183 | Warfarin | Methylprednisolone | 1 |  | 216 | Rivaroxaban | Enalapril | 1 |
| 184 | Warfarin | Medroxyprogesterone | 1 |  | 217 | Rivaroxaban | Erythromycin | 1 |
| 185 | Warfarin | Metronidazole | 1 |  | 218 | Rivaroxaban | Olmesartan | 1 |
| 186 | Warfarin | Menatetrenone | 1 |  | 219 | Rivaroxaban | Carbamazepine | 1 |
| 187 | Warfarin | Meloxicam | 1 |  | 220 | Rivaroxaban | Dipyridamole | 1 |
| 188 | Warfarin | Meropenem | 1 |  | 221 | Rivaroxaban | Cibenzoline | 1 |
| 189 | Warfarin | Ranitidine | 1 |  | 222 | Rivaroxaban | Selegiline | 1 |
| 190 | Warfarin | Ramatroban | 1 |  | 223 | Rivaroxaban | Doxazosin | 1 |
| 191 | Warfarin | Risperidone | 1 |  | 224 | Rivaroxaban | Nifedipine | 1 |
| 192 | Warfarin | Ribavirin | 1 |  | 225 | Rivaroxaban | Valsartan | 1 |
| 193 | Warfarin | Rifabutin | 1 |  | 226 | Rivaroxaban | Famotidine | 1 |
| 194 | Warfarin | Rifampicin | 1 |  | 227 | Rivaroxaban | Fenofibrate | 1 |
| 195 | Warfarin | Letrozole | 1 |  | 228 | Rivaroxaban | Fondaparinux | 1 |
| 196 | Warfarin | Lorazepam | 1 |  | 229 | Rivaroxaban | Heparinoid | 1 |
| 197 | Warfarin | An extract from inflammatory rabbit skin inoculated by vaccinia virus (Neurotrophin) | 1 |  | 230 | Rivaroxaban | Heparinoid sodium | 1 |
| 198 | Warfarin | Antipyretic analgesic | 1 |  | 231 | Rivaroxaban | Beraprost | 1 |
| 199 | Warfarin | Dried thyroid | 1 |  | 232 | Rivaroxaban | Rabeprazole | 1 |
| 200 | Warfarin | Enteral nutrient (detail unknown) | 1 |  | 233 | Rivaroxaban | Rivastigmine | 1 |
| 201 | Warfarin | Antibiotics | 1 |  | 234 | Rivaroxaban | Rosuvastatin | 1 |
| 202 | Warfarin | Psychotropic agents | 1 |  | 235 | Rivaroxaban | Coagulation inhibitor | 1 |
| 203 | Warfarin | Comprehensive cold medicine | 1 |  | 236 | Rivaroxaban | Unfractionated heparin | 1 |
| 204 | Warfarin | Antibiotics-resistant lactic | 1 |  | 237 | Dabigatran | Loxoprofen | 1 |
| 205 | Warfarin | Precipitated calcium carbonate. Cholecalciferol Magnesium | 1 |  | 238 | Dabigatran | Azosemide | 1 |
| 206 | Warfarin | Kampo Bakumondoto | 1 |  | 239 | Dabigatran | Amiodarone | 1 |
| 207 | Warfarin | non-pyrazolone anti-pyretics | 1 |  | 240 | Dabigatran | Imidafenacin | 1 |
| 208 | Warfarin | Butyrate-producing bacteria | 1 |  | 241 | Dabigatran | Candesartan | 1 |
| 209 | Warfarin | Itraconazole | 1 |  | 242 | Dabigatran | Digoxin | 1 |
| 210 | Warfarin | Erythromycin | 1 |  | 243 | Dabigatran | Febuxostat | 1 |
| 211 | Warfarin | Telithromycin | 1 |  | 244 | Dabigatran | Furosemide | 1 |
| 212 | Rivaroxaban | Celecoxib | 1 |  | 245 | Dabigatran | Phenytoin sodium | 1 |
| 213 | Rivaroxaban | Apixaban | 1 |  | 246 | Dabigatran | Rifampicin | 1 |
