# Supplementary material for: Prevalence of drug–drug interaction in atrial fibrillation patients based on a large claims data
Source: PLoS One. 2019 Dec 9;14(12):e0225297. doi: 10.1371/journal.pone.0225297 (PMC6901225; doi:10.1371/journal.pone.0225297)
Supplement: S2 Table — (DOCX) [file pone.0225297.s002.docx]

S2

| No. | Drug A | Drug B | inhibition and other mechanism | induction and other mechanism |  | No. | Drug A | Drug B | inhibition and other mechanism | induction and other mechanism |
| --- | --- | --- | --- | --- | --- | --- | --- | --- | --- | --- |
| 1 | Apixaban | Amiodarone | ☑ | □ |  | 26 | Apixaban | Rifampicin | □ | ☑ |
| 2 | Apixaban | Aprepitant | ☑ | □ |  | 27 | Apixaban | Rivaroxaban | ☑ | □ |
| 3 | Apixaban | Aspirin | ☑ | □ |  | 28 | Apixaban | Telithromycin | ☑ | □ |
| 4 | Apixaban | Aspirin-dialuminate | ☑ | □ |  | 29 | Apixaban | Ticlopidine | ☑ | □ |
| 5 | Apixaban | Aspirin-lansoprazole | ☑ | □ |  | 30 | Apixaban | Tofisopam | ☑ | □ |
| 6 | Apixaban | Bosentan | □ | ☑ |  | 31 | Apixaban | Verapamil | ☑ | □ |
| 7 | Apixaban | Carbamazepine | □ | ☑ |  | 32 | Apixaban | Voriconazole | ☑ | □ |
| 8 | Apixaban | Ciclosporin | ☑ | □ |  | 33 | Apixaban | Warfarin | ☑ | □ |
| 9 | Apixaban | Cilostazol | ☑ | □ |  | 34 | Edoxaban | Aspirin | ☑ | □ |
| 10 | Apixaban | Ciprofloxacin | ☑ | □ |  | 35 | Edoxaban | Aspirin-dialuminate | ☑ | □ |
| 11 | Apixaban | Clarithromycin | ☑ | □ |  | 36 | Edoxaban | Aspirin-lansoprazole | ☑ | □ |
| 12 | Apixaban | Clopidogrel | ☑ | □ |  | 37 | Edoxaban | Carbamazepine | □ | ☑ |
| 13 | Apixaban | Clopidogrel-aspirin | ☑ | □ |  | 38 | Edoxaban | Ciclosporin | ☑ | □ |
| 14 | Apixaban | Dabigatran | ☑ | □ |  | 39 | Edoxaban | Cilostazol | ☑ | □ |
| 15 | Apixaban | Diltiazem | ☑ | □ |  | 40 | Edoxaban | Clarithromycin | ☑ | □ |
| 16 | Apixaban | Edoxaban | ☑ | □ |  | 41 | Edoxaban | Clopidogrel | ☑ | □ |
| 17 | Apixaban | Erythromycin | ☑ | □ |  | 42 | Edoxaban | Clopidogrel-aspirin | ☑ | □ |
| 18 | Apixaban | Fluorouracil | ☑ | □ |  | 43 | Edoxaban | Dabigatran | ☑ | □ |
| 19 | Apixaban | Fluconazole | ☑ | □ |  | 44 | Edoxaban | Erythromycin | ☑ | □ |
| 20 | Apixaban | Imatinib | ☑ | □ |  | 45 | Edoxaban | Itraconazole | ☑ | □ |
| 21 | Apixaban | Itraconazole | ☑ | □ |  | 46 | Edoxaban | Quinidine | ☑ | □ |
| 22 | Apixaban | Miconazole | ☑ | □ |  | 47 | Edoxaban | Rifampicin | □ | ☑ |
| 23 | Apixaban | Phenobarbital | □ | ☑ |  | 48 | Edoxaban | Rivaroxaban | ☑ | □ |
| 24 | Apixaban | Phenytoin | □ | ☑ |  | 49 | Edoxaban | Telithromycin | ☑ | □ |
| 25 | Apixaban | Quinidine | ☑ | □ |  | 50 | Edoxaban | Ticlopidine | ☑ | □ |
| No. | Drug A | Drug B | inhibition and other mechanism | induction and other mechanism |  | No. | Drug A | Drug B | inhibition and other mechanism | induction and other mechanism |
| 51 | Edoxaban | Verapamil | ☑ | □ |  | 76 | Rivaroxaban | Bosentan | □ | ☑ |
| 52 | Edoxaban | Warfarin | ☑ | □ |  | 77 | Rivaroxaban | Carbamazepine | □ | ☑ |
| 53 | Dabigatran | Aspirin | ☑ | □ |  | 78 | Rivaroxaban | Ciclosporin | ☑ | □ |
| 54 | Dabigatran | Aspirin-dialuminate | ☑ | □ |  | 79 | Rivaroxaban | Cilostazol | ☑ | □ |
| 55 | Dabigatran | Aspirin-lansoprazole | ☑ | □ |  | 80 | Rivaroxaban | Ciprofloxacin | ☑ | □ |
| 56 | Dabigatran | Carbamazepine | □ | ☑ |  | 81 | Rivaroxaban | Clarithromycin | ☑ | □ |
| 57 | Dabigatran | Ciclosporin | ☑ | □ |  | 82 | Rivaroxaban | Clopidogrel | ☑ | □ |
| 58 | Dabigatran | Cilostazol | ☑ | □ |  | 83 | Rivaroxaban | Clopidogrel-aspirin | ☑ | □ |
| 59 | Dabigatran | Clarithromycin | ☑ | □ |  | 84 | Rivaroxaban | Diltiazem | ☑ | □ |
| 60 | Dabigatran | Clopidogrel | ☑ | □ |  | 85 | Rivaroxaban | Erythromycin | ☑ | □ |
| 61 | Dabigatran | Clopidogrel-aspirin | ☑ | □ |  | 86 | Rivaroxaban | Fluorouracil | ☑ | □ |
| 62 | Dabigatran | Erythromycin | ☑ | □ |  | 87 | Rivaroxaban | Fluconazole | ☑ | □ |
| 63 | Dabigatran | Itraconazole | ☑ | □ |  | 88 | Rivaroxaban | Imatinib | ☑ | □ |
| 64 | Dabigatran | Quinidine | ☑ | □ |  | 89 | Rivaroxaban | Itraconazole | ☑ | □ |
| 65 | Dabigatran | Rifampicin | □ | ☑ |  | 90 | Rivaroxaban | Miconazole | ☑ | □ |
| 66 | Dabigatran | Rivaroxaban | ☑ | □ |  | 91 | Rivaroxaban | Phenobarbital | □ | ☑ |
| 67 | Dabigatran | Telithromycin | ☑ | □ |  | 92 | Rivaroxaban | Phenytoin | □ | ☑ |
| 68 | Dabigatran | Ticlopidine | ☑ | □ |  | 93 | Rivaroxaban | Quinidine | ☑ | □ |
| 69 | Dabigatran | Verapamil | ☑ | □ |  | 94 | Rivaroxaban | Rifampicin | □ | ☑ |
| 70 | Dabigatran | Warfarin | ☑ | □ |  | 95 | Rivaroxaban | Telithromycin | ☑ | □ |
| 71 | Rivaroxaban | Amiodarone | ☑ | □ |  | 96 | Rivaroxaban | Ticlopidine | ☑ | □ |
| 72 | Rivaroxaban | Aprepitant | ☑ | □ |  | 97 | Rivaroxaban | Tofisopam | ☑ | □ |
| 73 | Rivaroxaban | Aspirin | ☑ | □ |  | 98 | Rivaroxaban | Verapamil | ☑ | □ |
| 74 | Rivaroxaban | Aspirin-dialuminate | ☑ | □ |  | 99 | Rivaroxaban | Voriconazole | ☑ | □ |
| 75 | Rivaroxaban | Aspirin-lansoprazole | ☑ | □ |  | 100 | Rivaroxaban | Warfarin | ☑ | □ |

| No. | Drug A | Drug B | inhibition and other mechanism | induction and other mechanism |  | No. | Drug A | Drug B | inhibition and other mechanism | induction and other mechanism |
| --- | --- | --- | --- | --- | --- | --- | --- | --- | --- | --- |
| 101 | Warfarin | Amiodarone | ☑ | □ |  | 123 | Warfarin | Imatinib | ☑ | □ |
| 102 | Warfarin | Aprepitant | ☑ | □ |  | 124 | Warfarin | Itraconazole | ☑ | □ |
| 103 | Warfarin | Aspirin | ☑ | □ |  | 125 | Warfarin | Lansoprazole | ☑ | □ |
| 104 | Warfarin | Aspirin-dialuminate | ☑ | □ |  | 126 | Warfarin | Lansoprazole-amoxicillin-clarithromycin | ☑ | □ |
| 105 | Warfarin | Aspirin-lansoprazole | ☑ | □ |  | 127 | Warfarin | Levofloxacin | ☑ | □ |
| 106 | Warfarin | Azithromycin | ☑ | □ |  | 128 | Warfarin | Menatetrenone | □ | ☑ |
| 107 | Warfarin | Bucolome | ☑ | □ |  | 129 | Warfarin | Miconazole | ☑ | □ |
| 108 | Warfarin | Capecitabine | ☑ | □ |  | 130 | Warfarin | Moxifloxacin | ☑ | □ |
| 109 | Warfarin | Carbamazepine | □ | ☑ |  | 131 | Warfarin | Norfloxacin | ☑ | □ |
| 110 | Warfarin | Celecoxib | ☑ | □ |  | 132 | Warfarin | Omeprazole | ☑ | □ |
| 111 | Warfarin | Ciclosporin | ☑ | □ |  | 133 | Warfarin | Pazufloxacin | ☑ | □ |
| 112 | Warfarin | Cilostazol | ☑ | □ |  | 134 | Warfarin | Phenobarbital | □ | ☑ |
| 113 | Warfarin | Ciprofloxacin | ☑ | □ |  | 135 | Warfarin | Phenytoin | □ | ☑ |
| 114 | Warfarin | Clarithromycin | ☑ | □ |  | 136 | Warfarin | Regorafenib | ☑ | □ |
| 115 | Warfarin | Clopidogrel | ☑ | □ |  | 137 | Warfarin | Rifampicin | □ | ☑ |
| 116 | Warfarin | Clopidogrel-aspirin | ☑ | □ |  | 138 | Warfarin | Roxithromycin | ☑ | □ |
| 117 | Warfarin | Erlotinib | ☑ | □ |  | 139 | Warfarin | Sunitinib | ☑ | □ |
| 118 | Warfarin | Erythromycin | ☑ | □ |  | 140 | Warfarin | Tegafur–gimeracil–oteracil | ☑ | □ |
| 119 | Warfarin | Fluorouracil | ☑ | □ |  | 141 | Warfarin | Tegafur–uracil | ☑ | □ |
| 120 | Warfarin | Fluconazole | ☑ | □ |  | 142 | Warfarin | Telithromycin | ☑ | □ |
| 121 | Warfarin | Garenoxacin | ☑ | □ |  | 143 | Warfarin | Ticlopidine | ☑ | □ |
| 122 | Warfarin | Iguratimod | ☑ | □ |  | 144 | Warfarin | Voriconazole | ☑ | □ |
