# Supplementary material for: Prevalence of drug–drug interaction in atrial fibrillation patients based on a large claims data
Source: PLoS One. 2019 Dec 9;14(12):e0225297. doi: 10.1371/journal.pone.0225297 (PMC6901225; doi:10.1371/journal.pone.0225297)
Supplement: S3 Table — (DOCX) [file pone.0225297.s003.docx]

| S3 | | | | |
| --- | --- | --- | --- | --- |
| ICD10 | Code | Japanese standard disease master | Code | The number of events |
| D500 | Iron deficiency anaemia secondary to blood loss (chronic) | 2809005 | hemorrhagic anemia | 26 |
| D62 | Acute posthaemorrhagic anaemia | 8832363 | acute hemorrhagic anemia | 11 |
| H113 | Conjunctival haemorrhage | 3727006 | subconjunctival hemorrhage | 21 |
| H313 | Choroidal haemorrhage and rupture | 3636002 | choroid rupture | 2 |
| H356 | Retinal haemorrhage | 3628025 | fundal hemorrhage | 12 |
| H356 | Retinal haemorrhage | 8840631 | retinal hemorrhage | 5 |
| H431 | Vitreous haemorrhage | 3792006 | vitreous hemorrhage | 24 |
| I609 | Subarachnoid haemorrhage, unspecified | 4309001 | subarachnoid hemorrhage | 17 |
| I610 | Intracerebral haemorrhage in hemisphere, subcortical | 4319027 | Brain subcortical hemorrhage | 5 |
| I610 | Intracerebral haemorrhage in hemisphere, subcortical | 4319030 | putaminal hemorrhage | 2 |
| I614 | Intracerebral haemorrhage in cerebellum | 4319006 | cerebellar hemorrhage | 3 |
| I615 | Intracerebral haemorrhage, intraventricular | 4319018 | intraventricular hemorrhage | 2 |
| I615 | Intracerebral haemorrhage, intraventricular | 4319032 | Hematoma Ventricular Perforation | 2 |
| I619 | Intracerebral haemorrhage, unspecified | 4319003 | hypertensive intracerebral hemorrhage | 5 |
| I619 | Intracerebral haemorrhage, unspecified | 4319020 | intracranial hemorrhage | 42 |
| I620 | Subdural haemorrhage (acute)(nontraumatic) | 4321008 | chronic subdural hematoma | 4 |
| I629 | Intracranial haemorrhage (nontraumatic), unspecified | 8839202 | Intracranial haemorrhage (nontraumatic) | 1 |
| I638 | Other cerebral infarction | 4341044 | hemorrhagic cerebral infarction | 5 |
| I841 | Other specified haemorrhoids | 8834643 | internal hemorrhoid hemorrhage | 3 |
| K250 | Gastric ulcer: Acute with haemorrhage | 5319011 | Gastric ulcer: Acute with haemorrhage | 7 |
| K254 | Gastric ulcer: Chronic or unspecified with haemorrhage | 8834632 | Gastric ulcer with haemorrhage | 35 |
| K260 | Duodenal ulcer: Acute with haemorrhage | 8845123 | Duodenal ulcer: Acute with haemorrhage | 1 |
| K290 | Acute haemorrhagic gastritis | 8834631 | haemorrhagic gastritis | 6 |
| K573 | Diverticular disease of large intestine without perforation or abscess | 8845806 | The ascending colon diverticular bleeding | 2 |
| K625 | Haemorrhage of anus and rectum | 8833703 | Haemorrhage of anus | 2 |
| K661 | Haemoperitoneum | 8839651 | hemoperitoneum | 1 |
| K921 | Melaena | 5781002 | lower gastrointestinal hemorrhage | 15 |
| K922 | Gastrointestinal haemorrhage, unspecified | 5789001 | gastric hemorrhage | 1 |
| K922 | Gastrointestinal haemorrhage, unspecified | 5789007 | gastrointestinal hemorrhage | 27 |
| K922 | Gastrointestinal haemorrhage, unspecified | 5789008 | upper gastrointestinal hemorrhage | 24 |
| K922 | Gastrointestinal haemorrhage, unspecified | 8837732 | intestinal hemorrhage | 2 |
| M2506 | Haemarthrosis | 9241024 | Hemarthrosis, knee | 3 |
| N368 | Other specified disorders of urethra | 5997011 | urethral hemorrhage | 1 |
| N921 | Excessive and frequent menstruation with irregular cycle | 6268001 | dysfunctional uterine bleeding | 1 |
| N921 | Excessive and frequent menstruation with irregular cycle | 8834262 | menometrorrhagia | 6 |
| N939 | Abnormal uterine and vaginal bleeding, unspecified | 6269007 | atypical genital bleeding | 5 |
| R040 | Epistaxis | 7847004 | epistaxis | 1 |
| R040 | Epistaxis | 8839467 | epistaxis syndorome | 20 |
| R048 | Haemorrhage from other sites in respiratory passages | 8838831 | pulmonary hemorrhage | 2 |
| R571 | Hypovolaemic shock | 9584004 | hemorrhagic shock | 93 |
| R58 | Haemorrhage, not elsewhere classified | 4590002 | hemorrhage | 12 |
| R58 | Haemorrhage, not elsewhere classified | 4590006 | internal hemorrhage | 2 |
| R58 | Haemorrhage, not elsewhere classified | 8832405 | acute major bleeding | 1 |
| S064 | Epidural haemorrhage | 8520005 | acute epidural hematoma | 1 |
| S064 | Epidural haemorrhage | 8520016 | epidural hematoma | 2 |
| S065 | Traumatic subdural haemorrhage | 8520015 | acute subdural hematoma | 2 |
| S0650 | Traumatic subdural haemorrhage | 8842898 | traumatic chronic subdural hematoma | 5 |
| S066 | Traumatic subarachnoid haemorrhage | 8520001 | traumatic subarachnoid hemorrhage | 1 |
| S068 | Other intracranial injuries | 8831759 | traumatic intracranial hemorrhage | 2 |
| S3680 | Injury of other intra-abdominal organs | 8844849 | traumatic intraabdominal hemorrhage | 3 |
| T810 | Haemorrhage and haematoma complicating a procedure, not elsewhere classified | 5258013 | tooth extraction hemorrhage | 1 |
| T810 | Haemorrhage and haematoma complicating a procedure, not elsewhere classified | 8833916 | secondary hemorrhage | 9 |
| T811 | Shock during or resulting from a procedure, not elsewhere classified | 8844477 | postoperative hemorrage shock | 2 |
