# Supplementary material for: Prevalence of drug–drug interaction in atrial fibrillation patients based on a large claims data
Source: PLoS One. 2019 Dec 9;14(12):e0225297. doi: 10.1371/journal.pone.0225297 (PMC6901225; doi:10.1371/journal.pone.0225297)
Supplement: S4 Table — (DOCX) [file pone.0225297.s004.docx]

| No. | Drug | Drug | Total (n) | Coadministration proportion (%) | Bleeding (n) | Bleeding rate (%) |
| --- | --- | --- | --- | --- | --- | --- |
| 1 | Warfarin | Miconazole | - | - | - | - |
| 2 | Rivaroxaban | Aspirin | 75 | 2.3 | 6 | 8.0 |
| 3 | Warfarin | Tegafur–gimeracil–oteracil | - | - | - | - |
| 4 | Warfarin | Aspirin | 252 | 7.7 | 56 | 22.2 |
| 5 | Rivaroxaban | Clopidogrel | 16 | 0.5 | 3 | 18.8 |
| 6 | Warfarin | Capecitabine | 1 | <0.1 | 0 | - |
| 7 | Warfarin | Tegafur–uracil | - | - | - | - |
| 8 | Warfarin | Lansoprazole | 159 | 4.8 | 38 | 23.9 |
| 9 | Warfarin | Celecoxib | 9 | 0.3 | 1 | 11.1 |
| 10 | Warfarin | Bucolome | 5 | 0.2 | 0 | 0 |
| 11 | Warfarin | Loxoprofen | 95 | 2.9 | 35 | 36.8 |
| 12 | Warfarin | Allopurinol | 104 | 3.2 | 4 | 3.8 |
| 13 | Warfarin | Levofloxacin | 8 | 0.2 | 0 | 0.0 |
| 14 | Warfarin | Amiodarone | 26 | 0.8 | 8 | 30.8 |
| 15 | Warfarin | Lornoxicam | 2 | <0.1 | 0 | 0 |
| 16 | Warfarin | Clarithromycin | 10 | 0.3 | 0 | 0 |
| 17 | Warfarin | Benzbromarone | 24 | 0.7 | 2 | 8.3 |
| 18 | Warfarin | Azithromycin | - | - | - | - |
| 19 | Warfarin | Omeprazole | 20 | 0.6 | 4 | 20.0 |
| 20 | Warfarin | Tramadol-Acetaminophen | 6 | 0.2 | 2 | 33.3 |
| 21 | Warfarin | Folinate | - | - | - | - |
| 22 | Warfarin | Erlotinib | - | - | - | - |
| 23 | Warfarin | Fluconazole | - | - | - | - |
| 24 | Warfarin | Rosuvastatin | 80 | 2.4 | 10 | 12.5 |
| 25 | Warfarin | Iguratimod | - | - | - | - |
| 26 | Warfarin | Gliclazide | 2 | <0.1 | 0 | 0 |
| 27 | Warfarin | Clopidogrel | 47 | 1.4 | 10 | 21.3 |
| 28 | Warfarin | Digoxin | 171 | 5.2 | 18 | 10.5 |
| 29 | Warfarin | Phenytoin | 3 | <0.1 | 1 | 33.3 |
| 30 | Warfarin | Sulfamethoxazole Trimethoprim | 6 | 0.2 | 0 | 0 |
| 31 | Warfarin | Ticlopidine | 4 | 0.1 | 0 | 0 |
| 32 | Warfarin | Verapamil | 96 | 2.9 | 4 | 4.2 |
| 33 | Warfarin | Voriconazole | - | - | - | - |
| 34 | Warfarin | Garenoxacin | 1 | <0.1 | 0 | 0 |
| 35 | Warfarin | Regorafenib | - | - | - | - |
| 36 | Warfarin | Prednisolone | 14 | 0.4 | 1 | 7.1 |
| 37 | Warfarin | Ciprofloxacin | - | - | - | - |
| 38 | Warfarin | Ezetimibe | 8 | 0.2 | 1 | 12.5 |
| 39 | Warfarin | Carbamazepine | 2 | <0.1 | 0 | 0 |
| 40 | Warfarin | Quetiapine | - | - | - | - |
| 41 | Warfarin | Pranlukast | 3 | <0.1 | 0 | 0 |
| 42 | Warfarin | Minocycline | 4 | 0.1 | 0 | 0 |
|  | | | | |  |  |

S4

Patients under poly-DDIs were duplicately counted in each DDI combinations.
